# A preliminary study of intensivist-performed DVT ultrasound screening in trauma ICU patients (APSIT Study)

**DOI:** 10.1186/s13613-020-00739-8

**Published:** 2020-09-14

**Authors:** Lloyd Roberts, Tom Rozen, Deirdre Murphy, Adam Lawler, Mark Fitzgerald, Harry Gibbs, Kyle Brooks, Joshua F. Ihle, Tim Leong, Judit Orosz, Eldho Paul, Vinodh Bhagyalakshmi Nanjayya

**Affiliations:** 1 Intensive Care Unit, Alfred Hospital, 55 Commercial Road, Melbourne, 3004 Australia; 2 Department of Epidemiology and Preventive Medicine, Monash University, Melbourne, 3004 Australia; 3 Royal Children’s Hospital, Melbourne, Australia; 4 Department of Paediatrics, University of Melbourne, Melbourne, Australia; 5 Murdoch Children’s Research Centre, Melbourne, Australia; 6 Vascular Laboratory, Alfred Hospital, Melbourne, 3004 Australia; 7 Australia Trauma Service, Alfred Hospital, Melbourne, 3004 Australia; 8 National Trauma Research Institute, Melbourne, 3004 Australia; 9 Department of General Medicine, Alfred Health, Melbourne, 3004 Australia; 10 Monash University, Melbourne, 3004 Australia; 11 Epworth HealthCare, Melbourne, Australia; 12 University of Melbourne, Melbourne, Australia

**Keywords:** Deep vein thrombosis, Compression ultrasound, Agreement, Vascular sonography, Trauma, Intensive care unit

## Abstract

**Background:**

Multiple screening Duplex ultrasound scans (DUS) are performed in trauma patients at high risk of deep vein thrombosis (DVT) in the intensive care unit (ICU). Intensive care physician performed compression ultrasound (IP-CUS) has shown promise as a diagnostic test for DVT in a non-trauma setting. Whether IP-CUS can be used as a screening test in trauma patients is unknown. Our study aimed to assess the agreement between IP-CUS and vascular sonographer performed DUS for proximal lower extremity deep vein thrombosis (PLEDVT) screening in high-risk trauma patients in ICU.

**Methods:**

A prospective observational study was conducted at the ICU of Alfred Hospital, a major trauma center in Melbourne, Australia, between Feb and Nov 2015. All adult major trauma patients admitted with high risk for DVT were eligible for inclusion. IP-CUS was performed immediately before or after DUS for PLEDVT screening. The paired studies were repeated twice weekly until the DVT diagnosis, death or ICU discharge. Written informed consent from the patient, or person responsible, or procedural authorisation, was obtained. The individuals performing the scans were blinded to the others’ results. The agreement analysis was performed using Cohen’s Kappa statistics and intraclass correlation coefficient for repeated binary measurements.

**Results:**

During the study period, 117 patients had 193 pairs of scans, and 45 (39%) patients had more than one pair of scans. The median age (IQR) was 47 (28–68) years with 77% males, mean (SD) injury severity score 27.5 (9.53), and a median (IQR) ICU length of stay 7 (3.2–11.6) days. There were 16 cases (13.6%) of PLEDVT with an incidence rate of 2.6 (1.6–4.2) cases per 100 patient-days in ICU. The overall agreement was 96.7% (95% CI 94.15–99.33). The Cohen’s Kappa between the IP-CUS and DUS was 0.77 (95% CI 0.59–0.95), and the intraclass correlation coefficient for repeated binary measures was 0.75 (95% CI 0.67–0.81).

**Conclusions:**

There is a substantial agreement between IP-CUS and DUS for PLEDVT screening in trauma patients in ICU with high risk for DVT. Large multicentre studies are needed to confirm this finding.

## Background

Deep vein thrombosis (DVT) is a common complication in adult trauma patients in ICU [1–3]. In the absence of prophylaxis, 40–80% of trauma patients develop DVT, and the risk persists even with prophylaxis [3–6]. Due to increased risk of bleeding, anticoagulation for DVT prophylaxis may be withheld for an extended period, particularly in patients with traumatic brain, pelvis, or spinal cord injuries. Guidelines recommend screening for DVT in patients who are unable to have prophylaxis [5, 7]. In clinical practice, DVT screening is performed using venous Duplex ultrasound (DUS), although contrast venography is the gold standard, as it avoids radiation and contrast exposure [8–11].

Generally, DUS is performed by vascular sonographers and reported by radiologists or vascular physicians. However, DUS is time-consuming, expensive, and there is an inherent delay in organising the test and obtaining a report [5, 12, 13]. As such, interest has grown in doctor-performed compression ultrasound for detecting DVT. Studies from the emergency department and outpatient settings have shown that 2- or 3-point compression ultrasound performed by doctors have similar diagnostic accuracy compared to DUS [14–16].

Published guidelines on general critical care ultrasound recommend that 2-point compression- (at the common femoral and popliteal veins) should be used by ICU physicians to diagnose DVT (grade 1B) [17, 18]. However, only two studies have been conducted in the ICU setting, and these studies did not include trauma patients [19, 20]. In both these studies, compression ultrasound was performed in patients with suspected DVT or pulmonary embolism. Also, in the study by Caronia et al. appropriate statistical techniques were not used to adjust for repeated scanning. Trauma patients differ from medical or surgical ICU or ED patients—their risk of DVT is high as pharmacological prophylaxis is withheld frequently. As a result, they require repeated screening scans to diagnose DVT. It is not clear whether compression ultrasound can be used as a screening test in this setting.

To evaluate the accuracy of compression ultrasound screening in ICU trauma patients at high risk of DVT, we conducted a prospective observational study comparing intensivist-performed compression ultrasound (IP-CUS) for diagnosing proximal lower extremity DVT (PLEDVT) to DUS. Our primary null hypothesis was that there was a fair agreement between the DUS and IP-CUS for the diagnosis of PLEDVT in high-risk trauma patients in ICU. In addition to this, we also wanted to evaluate the incidence of lower extremity DVT in high-risk trauma patients.

## Methods

We conducted this prospective study at the Alfred Hospital, a major trauma center in the state of Victoria in Melbourne, Australia, between Feb 2015 and Nov 2015. The Alfred intensive care unit is a 45-bed quaternary care unit in a 550-bed hospital. Annual ICU admissions are more than 3000 and nearly a third of them are following trauma.

We included consecutive major trauma (Injury Severity Score (ISS) > 12) adult patients (age ≥ 18 years) admitted to ICU who had at least one of the following issues: anticoagulation delayed (or expected to be delayed) for more than 2 days, spinal cord injury, leg fractures, pelvic fractures (other than isolated pubic ramus), major traumatic brain injury, underlying DVT risk factors (past history of venous thromboembolism, thrombophilic disorder, or active malignancy). We excluded patients with known DVT or in whom DUS was not possible (e.g., hindquarter amputation). Also, we excluded patients who had indicated not to be approached for studies in the health information services database, who had already enrolled in a study which did not allow co-enrolment in other studies, and in whom the treating clinicians felt enrolment was not in the best interest of the patients (e.g., open wounds, recent skin grafts, degloving injuries).

The detailed local guidelines used for thromboembolism prophylaxis is provided in Appendix. If pharmacological prophylaxis for DVT was contraindicated, sequential calf compression devices and graduate compression stockings were used according to ICU guidelines.

Patients were identified by reviewing ICU and trauma units’ electronic databases and admission notes. A vascular lab sonographer performed lower extremity DUS within 96 h of admission and twice weekly after that while in ICU. The DUS included compression, color Doppler and pulsed wave Doppler for the diagnosis of DVT. The study was performed using Toshiba Aplio™ MX, 400 or 500 ultrasound machines (Toshiba Medical Systems Co Ltd, Otawara, Japan) using a 5.8–7.6 MHz linear probe. A vascular imaging specialist (vascular surgeon or vascular physician) reviewed the scans and reported on the scans.

**Fig. 1:**
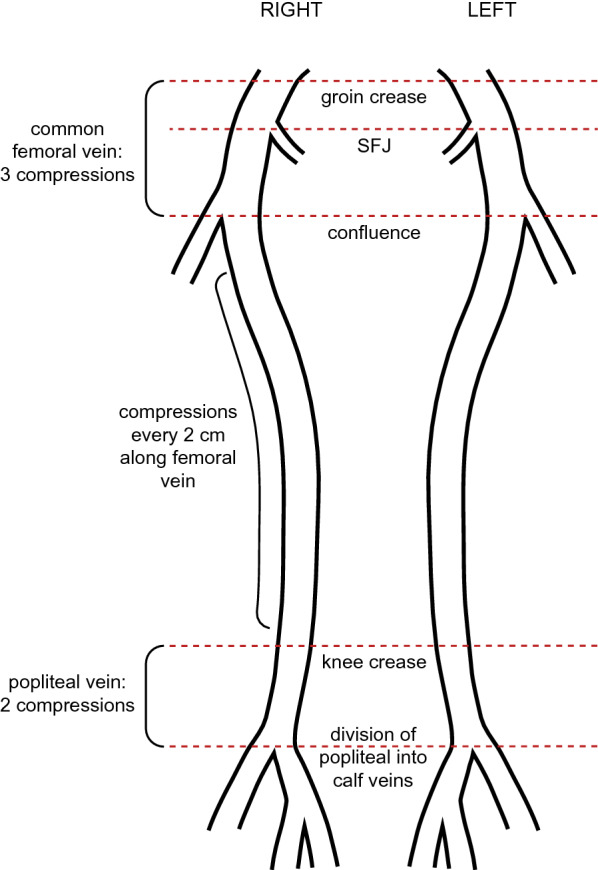
Schematic diagram of deep veins in the lower limbs showing the compression points. The intensivist-performed compression ultrasound scan protocol consisted of compression of common femoral, femoral, and popliteal veins. Common femoral vein compression was performed at groin crease, saphenofemoral junction (SFJ) and confluence of femoral and deep femoral veins. Femoral vein compression was performed at 2 cm intervals along the vein below the confluence and popliteal vein compression was performed at the groin crease and the confluence of calf veins

**Fig. 2:**
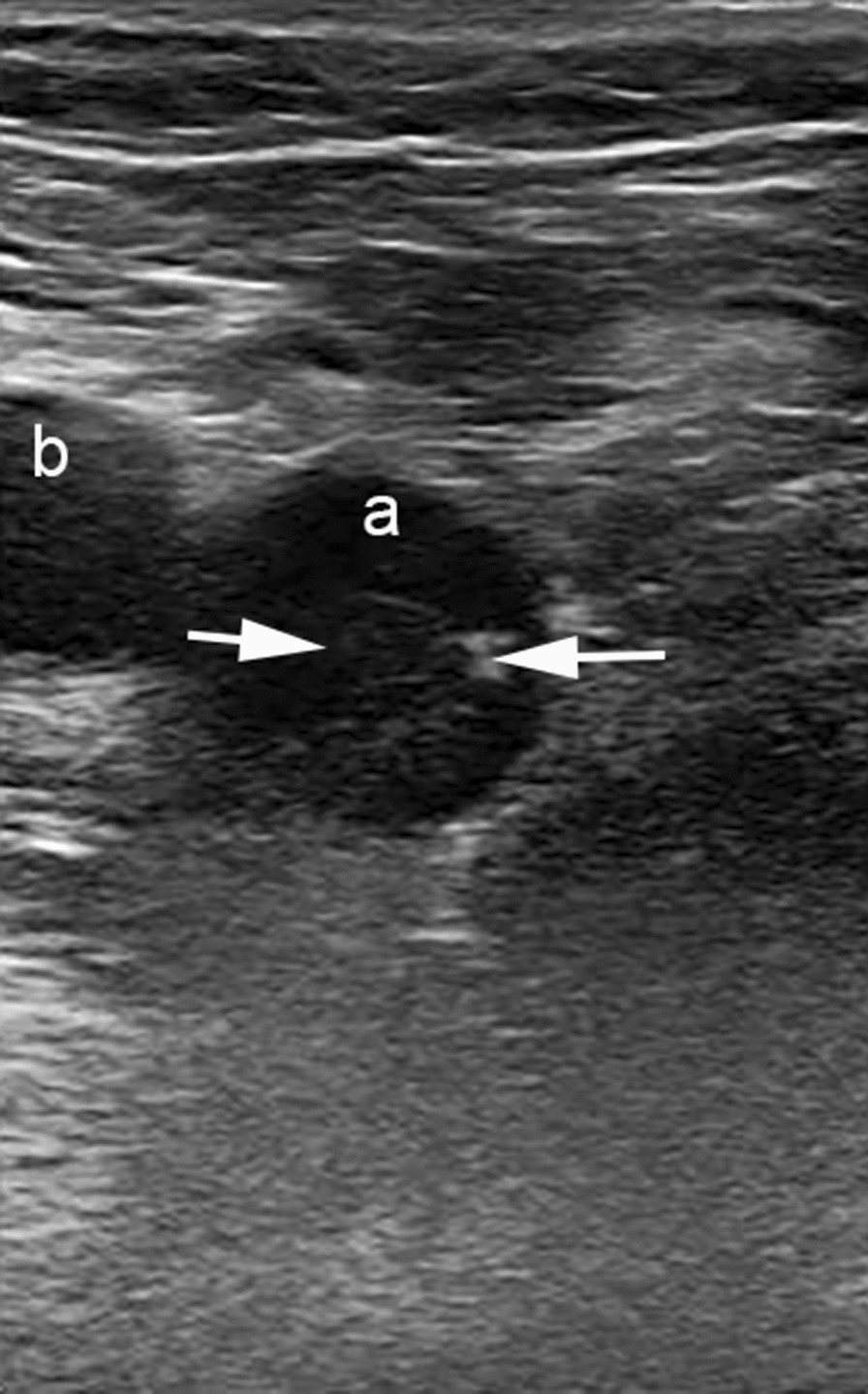
Ultrasound image of a person with compression ultrasound confirmed DVT. Short axis ultrasonographic view of the right femoral vein (**a**) showing thrombus (arrows) attached to the wall. The right superficial femoral artery (**b**) is seen adjacent to the femoral vein. DVT: deep vein thrombosis

The IP-CUS was performed immediately before or after DUS using a Philips^®^ Sparq (Philips Ultrasound, Bothell, WA, USA) machine and broadband linear array transducer (L12-4) of 4–12 MHz frequency with a standard scan setting. We used the IP-CUS technique described by Kory et al. to assess proximal veins [19]. The scan consisted of compression of the common femoral vein at 3 points (groin crease, saphenofemoral junction and the confluence of femoral and deep femoral veins), compression of the femoral vein at 2 cm intervals and compression of the popliteal vein at 2 points (knee crease and division of popliteal vein to calf veins) (Fig. 1). The ICU physician applied compression by the ultrasound probe until the complete apposition of the vein walls was visualised or deformation of the accompanying artery without vein collapse, suggesting the presence of a DVT. If there was a visible thrombus, no compression was applied to the vein (Fig. 2). Before the IP-CUS, the ICU physician tilted the bed 30 degrees in a reverse Trendelenburg position and externally rotated the leg, if not contraindicated. For each pair of IP-CUS and DUS, both operators were blinded to the other’s results at the time of the scan. If there was a discrepancy in the results, the vascular sonographer repeated DUS on the same day and the vascular imaging specialist adjudicated the results. Adjudicated results from the vascular imaging specialist were used in the final analysis of the agreement. The operator notified the results of the scan to the treating ICU team only where even a short delay in disclosure would have caused harm (e.g., a mobile tongue of thrombus seen). Otherwise, they received the reports as usual from the vascular laboratory on the electronic medical records. The treating physician decided treatment for DVT and further investigations like CTPA. Patients continued to have bi-weekly paired scans until they developed DVT, died, or were discharged from the ICU.

Nine ICU physicians (five consultants and four critical care ultrasound fellows) performed IP-CUS. Of these, four had a Diploma of Diagnostic Ultrasound (DDU (Critical Care)), the peak qualification for critical care ultrasound in Australia and New Zealand, and five had enrolled in DDU. DDU holders and trainees had ≥ 4 and 2–3 years of experience in performing critical care ultrasound, respectively. The centre is a training centre for DDU (Critical Care) and the training curriculum for DDU (Critical Care) includes diagnosis of DVT using 2D, Colour Doppler and Puled Wave Doppler [21]. For the purpose of the study, the ICU physicians were only allowed to use compression ultrasound. They could use Colour Doppler to localise and identify vessels if required, but for no other purposes. All the ICU physicians received “one” hour session prior to the study commencement to familiarise them with the study protocol.

Along with the results of the scans, patient demographics, illness severity score, and data related to trauma, ICU, and hospital stay were collected.

### Outcomes

The primary outcome was the agreement of IP-CUS with DUS for the detection of PLEDVT. Secondary outcomes included sensitivity and specificity analysis, incidence, and incidence rate of both proximal and distal lower limb DVT.

### Ethics and consent

Alfred Hospital’s ethics committee approved the study (10/14). Informed consent was obtained before ultrasound examination and data collection from the patient or next of kin where possible; otherwise, enrolment was permitted via procedural authorisation under the Guardianship and Administration Act 1986.

### Statistical analysis

Continuous variables were summarised using the mean and standard deviation or median with 25th and 75th quartiles according to data type and distribution. Categorical variables were expressed as counts and percentages. Cohen’s Kappa was used to summarise the agreement between IP-CUS and DUS to diagnose PLEDVT. The 95% confidence interval was calculated using the standard error calculation suggested by Fleiss. The interpretation of Kappa was based on the classification by Landis and Koch [22]. To account for the repeated ultrasound examinations in the same patient, further analysis was performed by fitting a linear mixed model with operator (ICU physician or vascular sonographer), scan number, and operator by scan number as fixed effects; patient, patient by scan number interaction as random effects, and calculated intraclass correlation coefficient for binary repeated measurement [23, 24]. Statistical analysis was conducted using Stata SE 15.0 (StataCorp, Tx, USA) and SAS 9.4 (SAS Institute, Cary NC).

## Results

One hundred and seventeen patients had 193 pairs of scans performed during the study period. Forty-six (39%) patients had more than one pair of DVT scans performed during the study period. Seven scans reported by ICU physicians as uncertain for DVT and two repeat scans performed in patients who were already diagnosed to have DVT in their previous scans (protocol violation) were excluded. Therefore, 184 scans from 117 patients were included in the final analysis of the agreement.

Table 1 shows the patient demographics, illness severity scores, DVT prophylaxis, and other ICU characteristics. The study population consisted predominantly of males (77%) with a median (IQR) age of 47 (28–68) years. Traumatic brain injury (TBI) was the most common type of injury (50%). The mean (SD) ISS was 27.5 (9.53) and the median (IQR) Acute Physiology and Chronic Health Evaluation (APACHE) II score was 14 (10–19). The main risk factor for DVT was the presence of femoral central venous cannula (CVC) which was present in 26 patients (22%). Calf compression devices or compression stockings were the most common types of physical adjuncts used for DVT prophylaxis. Nearly 75% of the patients were on mechanical ventilation-median (IQR) duration of 5.4 (2.4–9.5) days. Most of the patients (104/117 (89%)) survived the hospital stay with a median (IQR) ICU length of stay of 6.6 (3.2–11.6) days.

**Table 1:** Demographic and Clinical Characteristics of the patients in the study

Patient characteristics		All patients (N = 117)
Age	Median (IQR) years	47 (28–68)
Sex-male	*n* (%)	100 (77)
Trauma type		
TBI	*n* (%)	58 (50)
Pelvic/lower limb fracture	*n* (%)	32 (27)
Spinal cord injury	*n* (%)	10 (9)
Risk factor for DVT		
Known h/o DVT/PE	*n* (%)	4 (3)
Femoral CVC present	*n* (%)	26 (22)
Location of femoral CVC-right	*n* (%)	23 (88)
Severity of Illness Scores		
ISS score	Mean (SD)	27.5 (9.53)
APACHE II Score	Median (IQR)	14 (10–19)
DVT prophylaxis		
Calf compression devices	*n* (%)	103 (88)
Compression stockings	*n* (%)	106 (91)
Enoxaparin**	*n* (%)	13 (11)
Mechanical ventilation	*n* (%)	86 (74)
Duration of mechanical ventilation	Median (IQR) days	5.4 (2.4–9.5)
ICU length of stay	Median (IQR) days	6.6 (3.2–11.6)
Hospital length of stay*	Median (IQR) days	17 (11–22)
Survival	*n* (%)	104 (89)

*IQR* interquartile rage, *SD* standard deviation, *TBI* traumatic brain injury, *DVT* deep vein thrombosis, *PE* pulmonary embolism, *CVC* central venous catheter, *ISS* injury severity score, *APACHE II* Acute Physiology and Chronic Health Evaluation II

*Data missing for 2 patients

**1 patient received unfractionated heparin

The median (IQR) number of studies conducted by the ICU physicians were 15 (8–35) scans. The median (IQR) time to first scan was 2 (1–5) days. The mean (SD) time for the IP-CUS scan was 8.8 (3.13) minutes. The vascular sonographer performing DUS was a senior vascular sonographer. Mean (SD) time for the DUS, including proximal and distal veins, was 12.8 (4.12) minutes. The mean difference in duration between the scans performed by the ICU physicians and the vascular sonographer was 4.1 min (95% CI 3.38–4.76; *p* < 0.01). However, the DUS time includes diagnosis of both PLEDVT and distal vein DVT.

Both IP-CUS and DUS revealed 12 DVTs in 184 scans. However, a discrepancy was noted in 5 scans—ICU physicians had diagnosed DVT which the DUS didn’t confirm. Adjudication ratified 4/5 (80%) of these scans as having DVT. These DVTs were mainly associated with femoral CVC. All the diagnosed DVT were asymptomatic. Table 2 summarises the results of the scans performed by the ICU physicians and the vascular sonographer after adjudication. Thus, the incidence of PLEDVT was 13.6% (16/117) with an incidence rate of 2.6 (95% CI 1.61–4.28) cases per 100 patient-days. There were 13 cases which had distal lower limb DVT diagnosed by DUS, of which three cases also had a PLEDVT. Therefore, the incidence of lower extremity DVT was 22.2% (26/117) with an incidence rate of 4.4 (95% CI 3.08–6.55) cases per 100 patient-days. The median (IQR) time to the occurrence of PLEDVT was 3.5 (2.44–7.09) days.

**Table 2:** Cross tabulation of intensivist-performed compression ultrasound findings with the adjudicated findings on Doppler ultrasound

	IP-CUS results for DVT	
Adjudicated Doppler US results for DVT	Positive	Negative
Positive	11	5
Negative	1	167

*IP-CUS* intensivist-performed compression ultrasound, *DVT* deep vein thrombosis, *US* ultrasound

Out of the sixteen patients who had PLEDVT, five patients received IVC filter and five patients had therapeutic anticoagulation with heparin or enoxaparin. Rest of the patients were followed up using DUS and did not receive any treatment for their DVT. Two patients (1.7%) developed pulmonary embolism (PE) and both were segmental PE. Amongst the thirteen patients diagnosed with distal lower extremity DVT, only two patients received full dose anticoagulation. Rest of the patients received prophylactic dose of enoxaparin along with follow-up DUS. Three patients had IVC filter to prevent PE. None of the patients in the distal lower extremity DVT group developed PE. No deaths occurred due to PE in the current study.

### Characteristics of DVT on DUS

Of the 16 patients with DVT, 11 (69%) had right-sided DVT, and seven (44%) had CVC associated thrombus. The median (IQR) length was 2 (2–5) cm. The most common location of DVT was the common femoral vein (13 cases (81%)) followed by femoral (4 cases (25%), profunda femoris (2 cases (12.5%), and external iliac (2 cases (12.5%) veins. Four cases (25%) had DVT in more than one venous segment. Nearly all the thrombi (94%) were fixed and non-occlusive.

### Agreement between the ICU physician and vascular sonographer

After adjudication, IP-CUS findings were concordant with DUS in 178 of 184 scans (Percent agreement (95% CI): 96.7 (94.15–99.33)). The Cohen’s Kappa for the agreement was 0.77 (95% CI 0.59–0.95), suggesting substantial agreement between ICU physicians and Vascular sonographers concerning the diagnosis of DVT. For the unadjudicated results from the initial vascular sonographer DUS, the Cohen’s Kappa was 0.55 (0.30–0.80) suggesting moderate agreement. The intraclass correlation coefficient calculated using a linear mixed model to account for repeated measurements was 0.76 (0.69–0.81) which suggested good agreement between the two methods.

The misclassification occurring in the six scans were due to 5 false-negative and one false-positive scan leading to a sensitivity of 69% (95% CI 41.3–89), specificity of 99% (95% CI 96.7–100), positive predictive value of 92% (60.3–98.8) and negative predictive value 97% (94.2–98.6). All the false-negative cases had non-occlusive DVT—3 cases (60%) in common femoral vein and 2 cases (40%) in the femoral vein. Three scans (60%) were repeated scans in the same patients, and three scans (60%) had CVC associated thrombus. In one patient, the scan was commented to be “difficult to perform” by the intensivist due to extensive lower limb injury and positioning restriction. The five false-negative scans were missed by four ICU physicians- each missing one case and one physician missing two cases. The false-positive scan was due to the misinterpretation of the femoral artery as a vein at the left saphenofemoral junction.

## Discussion

To our knowledge, this is the first prospective observational study from a major trauma center which looked at the agreement between IP-CUS and DUS in the setting of DVT screening. We found a substantial agreement between these two methods (Cohen’s Kappa 0.77 (95% CI 0.59–0.95) and ICC for binary repeated measurements 0.76 (95% CI 0.69–0.81)).

Two previous ICU studies have looked at the question of agreement between intensive care physicians, and vascular sonographers performed DUS. Both of them included patients in whom DVT diagnosis or PE was suspected. The first study by Kory et al., a multicentre study in medical, surgical and cardiothoracic ICUs in patients with suspected DVT, PE, or both, compared the agreement between the intensive care physicians trained in critical care ultrasound with formal vascular DUS [19]. In 128 paired scans, they found a sensitivity of 86% and specificity of 96% with a diagnostic accuracy of 95%. In the second study by Caronia et al., a single-centre study in a medical ICU or intermediate care unit in patients with suspected DVT, agreement of 2 point compression ultrasound performed by medical residents trained in a 2-hrs module in focused vascular sonography was compared with DUS [20]. The reported sensitivity of 63% was low and Caronia et al. concluded that 2-point compression ultrasound was inadequate to diagnose DVT.

Our result of 69% sensitivity is comparable to the study by Caronia et al. However, their study used a 2-point compression method in which compression was only applied to common femoral and popliteal veins. As a result, their study missed femoral vein DVT leading to lower sensitivity. In contrast, we used the protocol suggested by Kory et al., which included compression of the femoral vein along with common femoral and popliteal veins. We noted that all the cases missed by IP-CUS had non-occlusive DVT and were located either in common femoral or femoral veins. Most of the patients had CVC associated DVT. It is interesting to note that vascular DUS missed four cases of DVT and three of these cases also had CVC associated DVT. In the study by Kory et al., femoral CVC sites were excluded and the study by Caronia et al. does not mention about CVC at the site of scanning. Similarly, none of the previous ED and outpatient studies had patients with femoral CVC. We can only speculate that the presence of femoral CVC might have made it harder to do the IP-CUS and the incompressibility at the site of DVT might have been attributed to CVC rather than DVT by the operators leading to the reduced sensitivity of IP-CUS for the diagnosis of DVT.

In terms of the epidemiology of DVT in trauma patients, our study is comparable to study by Hamada et al. They looked at the incidence and risk factors for DVT using a DUS screening method which used 2D, Colour and Continuous Wave Doppler in both upper and lower limbs of all consecutive trauma patients [3]. In our study, we found a mean incidence of 4.4 cases per 100 patient days (30.8% patients-week) for lower limb DVT. This incidence is higher than the mean incidence of 18% patients-week reported by Hamada et al. This may be because our study included adult major trauma patients at high risk of DVT, majority of whom could not receive pharmacologic prophylaxis. This reason may also explain the median time for the appearance of DVT of 3.5 days in our study which is much earlier than the median time of 6 days reported by Hamada et al. Similar to the findings of Hamada et al. we found more DVTs at the site of CVC insertion.

One of the main issues with the conduct of the study was that the gold standard test for diagnosing DVT, contrast venogram, is not readily available. As a result, most studies in both ED and ICU settings have used results of sonographer performed, and radiologist reported DUS as the gold standard for the diagnosis of DVT. However, as we have shown in this study, DUS can also miss DVT. If we did not have an adjudication step in our study, the sensitivity and specificity of our study would have been much lower. Therefore, using sensitivity and specificity, which is the test applied when a new test is being compared to the gold standard test, is not the right approach. The main method of agreement when the gold standard is not available is Cohen’s Kappa. Only few studies in ED and ICU settings have reported Kappa statistics [20, 25, 26].

### Strengths of the study

We used a standardised protocol with a standard definition for identifying high-risk trauma patients for DVT and recruited consecutive patients to minimise bias. Biweekly scanning occurred on standard weekdays and the paired scans were performed immediately after each other to minimise discordance due to embolisation or development of DVT. The ICU clinicians performing IP-CUS studies included experienced consultants and ultrasound Fellows with more than 1 year of scanning experience. We also had an adjudication step when there was a discrepancy in the finding of DVT between the ICU clinician and the DUS report. We allowed for repeated scanning, as patients admitted in ICU following trauma would need multiple scans during their admission to screen for DVT. Also, unlike previous studies, we adjusted for repeated measurements in the analysis to obtain a precise estimate of the agreement.

### Limitations

Our study has certain limitations. This was a single-centre study. However, the study was done in a population of trauma patients who were at high risk of DVT which makes it applicable to other trauma units treating similar patient groups. We did not look at the agreement for distal lower limb DVT. Hence, the results cannot be extrapolated to include distal lower limb DVT. The incidence of DVT was low and type 2 error as a result of the small sample size is possible. However, on posthoc analysis, we found that 180 paired scans were sufficient to detect a statistically significant Kappa (*p* ≤ 0.05) with 80% power and assuming the null hypothesis value of Kappa to be 0.40 [27]. Intra-rater reliability for diagnosis of DVT could not be assessed in our study, as repeating IP-CUS in a patient who has a diagnosis of DVT might risk pulmonary embolism. The overall incidence of DVT is probably higher than the one reported in our study, because we did not look for proximal upper limb DVT (e.g., jugular veins, subclavian veins).

## Conclusion

There is a substantial agreement between the IP-CUS compared to the vascular sonographer DUS for screening for DVT in multi-trauma patients at high risk. The discrepancy was noted mainly in patients who had central venous lines at the site of the scan. Large multicentre studies are needed to confirm these findings in the future.

## Data Availability

The dataset used and analysed during the current study is available from the corresponding author on reasonable request.

## Appendix

### Guidelines for venous thromboembolism prophylaxis in trauma

Venous thromboembolism prophylaxis in trauma patients is managed using the following guidelines in ICU.

### Pharmacological prophylaxis

- Prophylactic enoxaparin is commenced within 24 h of admission, unless contraindicated, in all patients, and is uncommon to be delayed for more than 7 days without compelling reasons including high risk or active bleeding.
  - Standard prophylaxis is enoxaparin 40 mg subcutaneous daily
  - For patients with low body weight (< 50 kg) or renal impairment (creatinine clearance < 30 mL/min) the dose of enoxaparin is 20 mg subcutaneous daily
  - For patients with high body weight (> 130 kg) the dose is enoxaparin 60 mg daily or 40 mg twice daily.
- In patients with traumatic brain injury, the time of pharmacological prophylaxis is discussed with the neurosurgeon. Provided there is no significant coagulopathy, thrombocytopenia, and no other contraindications:
  - If the head CT shows no obvious haemorrhages, enoxaparin may be started after a normal progress CT scan at 24–48 h
  - If the head CT shows small petechial haemorrhages only and a progress CT scan shows no evolution, then enoxaparin may be started at 48–72 h
  - If the head CT shows any significant haemorrhages (extradural, subdural, intraparenchymal, intraventricular, subarachnoid), enoxaparin should be delayed but usually started no later than 7 days.
- In patients with spinal column fractures or spinal cord injuries including epidural haematomas, pharmacological prophylaxis is discussed with the relevant orthopaedic or neurosurgical team
- In patients with ocular injuries pharmacological prophylaxis is discussed with Ophthalmologist
- In patients with solid organ injuries, e.g., liver, splenic or renal lacerations, prophylaxis is discussed with the Trauma surgeon and is generally delayed by about 48 h
- Enoxaparin is withheld on the day of surgery or procedure unless it is specifically ordered by the medical officer
- Enoxaparin is routinely administered 6 h post-surgery, unless specified otherwise by the medical officer.

### Mechanical prophylaxis

- Unless contraindicated, trauma patients in ICU have both mechanical and chemical prophylaxis.
- Intermittent pneumatic compression devices (IPC) and Graduated compression stockings (GCS) was recommended as an adjunct to the prevention of deep vein thrombosis (DVT) at the time of the study.
- When pharmacological prophylaxis was contraindicated in trauma patients, IPC and GCS was used, unless contraindicated, during the study period.
- Currently, only IPC is recommended. GCS is recommended only when IPC and pharmacological prophylaxis is contraindicated.

### Inferior vena cava (IVC) filter

- After discussion between the Trauma consultant and ICU consultant and weighing the risks vs benefits, IVC filter may be considered in the following high-risk trauma patients in whom pharmacological prophylaxis is contraindicated for more than 72 h to prevent pulmonary embolism
  - Spinal injury
  - Pelvic fractures or multiple lower limb long bone fractures.

### Serial surveillance ultrasound for DVT

- Patients with a contraindication to pharmacological prophylaxis and a contraindication to mechanical prophylaxis on at least one leg require serial surveillance Duplex ultrasound scans of the lower limbs to evaluate for asymptomatic DVT.

## Abbreviations

APACHE: Acute Physiology and Chronic Health Evaluation
CI: Confidence interval
CVC: Central Venous Catheter
DUS: Duplex ultrasound scans
DVT: Deep vein thrombosis
ED: Emergency Department
ICU: Intensive care unit
IP-CUS: Intensive care physicians performed compression ultrasound
IQR: Interquartile range
ISS: Injury Severity Score
PLEDVT: Proximal lower extremity deep vein thrombosis
SD: Standard deviation
TBI: Traumatic brain injury
